# NanoStriDE: normalization and differential expression analysis of NanoString nCounter data

**DOI:** 10.1186/1471-2105-12-479

**Published:** 2011-12-16

**Authors:** Christopher D Brumbaugh, Hyunsung J Kim, Mario Giovacchini, Nader Pourmand

**Affiliations:** 1 Department of Biomolecular Engineering, University of California at Santa Cruz, Santa Cruz, CA 95064, USA

## Abstract

**Background:**

The nCounter analysis system (NanoString Technologies, Seattle, WA) is a technology that enables the digital quantification of multiplexed target RNA molecules using color-coded molecular barcodes and single-molecule imaging. This system gives discrete counts of RNA transcripts and is capable of providing a high level of precision and sensitivity at less than one transcript copy per cell.

**Results:**

We have designed a web application compatible with any modern web browser that accepts the raw count data produced by the NanoString nCounter analysis system, normalizes it according to guidelines provided by NanoString Technologies, performs differential expression analysis on the normalized data, and provides a heatmap of the results from the differential expression analysis.

**Conclusion:**

NanoStriDE allows biologists to take raw data produced by a NanoString nCounter analysis system and easily interpret differential expression analysis of this data represented through a heatmap. NanoStriDE is freely accessible to use on the NanoStriDE website and is available to use under the GPL v2 license.

## Background

In recent years, RNA expression studies have relied on two major technologies: microarrays and high-throughput sequencing. Although both of these methods have proven their utility in biological assays [1,2], each has its limitations. Microarray analyses offer low cost, transcriptome-wide assays, but are hindered by a low dynamic range of detection [3,4]. RNA-Seq experiments offer greater sensitivity and digital measurements of transcript abundance, but require complex sequence analysis and have a relatively high cost [5,6]. The NanoString nCounter is a novel RNA-based technology whose costs and abilities place it firmly between microarrays and RNA-Seq [7].

The nCounter allows digital quantification of multiplexed target molecules through the use of color-coded molecular barcodes and a single-molecule imaging system [8]. By providing discrete counts of RNA transcripts, the nCounter overcomes the saturation limitations of microarrays while avoiding the complex sequence analysis necessitated by RNA-Seq. The platform can quantify up to 800 different RNA targets simultaneously in up to 12 samples per run, making the system ideal for studying differential expression and microRNA expression assays. Hands-on time for the assay is under one hour and the full process from input RNA to data output can be completed in one to two days.

Differential expression analysis is performed differently for microarrays and RNA-Seq because of distinctions in the underlying technologies. Microarrays measure expression levels using optical detection of fluorescent intensities. Two-sided t-tests are normally used with microarray data as log normalized fluorescent intensity levels are well modelled by the Gaussian distribution. RNA-Seq uses sequence coverage as a measurement of expression, producing discrete rather than continuous data. The use of a discrete distribution like the Poisson, or more appropriately the negative binomial [9,10], is appropriate in this case. The NanoString nCounter is most similar to RNA-Seq in that it processes discrete counts of measurement similar to RNA-Seq; as such, it is more appropriate to utilize differential expression analysis tools developed for RNA-Seq for data generated by the NanoString nCounter. For historical reasons and to allow comparisons, our server allows for both t-tests as well as a negative binomial-based test for the discovery of differentially expressed genes, a feature that allows us to offer an array of options in the web-based data analysis package described below.

Although the technology overcomes major limitations of microarrays and high throughput sequencing, the data produced by the NanoString nCounter is not in a directly usable format and must be compiled, normalized, and analyzed using a variety of statistical methods. Guidelines to achieve this are provided by the company; however, the process is complex and generally requires the use of Microsoft Excel running on a Windows system. Moreover, performing statistical analysis of the data to explore relevant trends and produce differential expression maps requires familiarity with R and associated packages like Bioconductor and DESeq. The use of this software and an understanding of which analyses must be performed and in what order is not part of most biologists' skill sets and so acts as a needless impediment to their effective use of nCounter data.

Our NanoString web application, NanoString Differential Expression (NanoStriDE), provides a configurable, extremely simple-to-use interface as a front end to a fully automated analysis pipeline of R libraries and Perl scripts. nCounter data is uploaded directly to the website. Processed data with a differential expression heatmap is made available for download within minutes.

## Implementation

### Controls and normalization

NanoStriDe intelligently applies positive and negative corrections, as well as a sample content normalization as per manufacturer guidelines to the raw data. The first stage applied to the data is a correction to positive controls. Each NanoString experiment contains synthetic spike-in controls in the early stage preparatory mix that allow for the correction of sample-to-sample variation due to assay-specific factors such as differences in amount of input material or reagents. The positive correction is calculated by

(1) $$c\times \left(\frac{m}{s}\right)$$

where *c *is count data for a gene in a given sample, *m *is the mean of the sum of the positive controls across all samples, and *s *is the sum of all of the positive controls for that given sample. The positive correction is applied to the data only if the t-test or one-way ANOVA was chosen. DESeq and one-way ANOVA (negative binomial) using DESeq ANODEV uses DESeq's built in normalization methods.

After the positive correction is applied, one of four negative correction methods is employed. The negative correction subtracts background noise from the positively-corrected data using counts of sequence tags known to be absent from the assay. The first three choices of negative correction use: (a) the mean of the negative controls for a given sample, (b) the mean of the negative controls summed with 2 standard deviations of the negative controls, or (c) the maximum of the negative controls. For these choices, the negative control is applied by

(2) $$\left\{\begin{array}{cc} c-n & \text{for}\;c-n\geq 0\\ 0 & \text{for}\;c-n<0 \end{array}\right)$$

where *c *is the count data and *n *is one of the values for the aforementioned negative controls. The fourth choice of negative control that can be applied is a one-tailed Student's t-test using the negative controls as one group against all samples of a given gene as the other group, and using this to determine the significance of a given gene with a p-value cutoff. This fourth type of negative correction is applied by

(3) $$\left\{\begin{array}{cc} c-m & \text{for}\;\text{p-value}<p\;\text{and}\;\left(c-m\right)\geq 0\\ 0 & \text{for}\;\text{p-value}<p\;\text{and}\;\left(c-m\right)<0\;\text{or}\;\text{for}\;\text{p-value}\;\geq p \end{array}\right)$$

where *c *is all count data for a given gene, *m *is the mean of the count data for that gene across all samples, and *p *is the p-value cutoff used. If the p-value is at or above the provided cutoff (default 0.05), the counts for the gene are not significant and are set to 0 for all samples. If the p-value is below the provided cutoff, the mean for the count data for the gene are subtracted from the counts for the gene and count values that fall below zero are set to zero. As with the positive correction, the negative correction is applied to the data only if the t-test or one-way ANOVA was chosen. DESeq and one-way ANOVA (negative binomial) using DESeq ANODEV uses DESeq's built in normalization methods.

The normalization employed after the positive and negative correction steps depend on the type of statistical analysis selected. If DESeq or one-way ANOVA (negative binomial) was chosen for the differential expression, then the normalization process used within DESeq is applied to the data and the sample content normalization is not applied to the data as application of the sample content normalization and the DESeq normalization would be redundant. Refer to DESeq for the details of the normalization process applied [10]. If the t-test or either one-way ANOVA is chosen for the differential expression, then one of three sample content normalization choices is applied:

*Option 1*: Normalize to the mRNA housekeeping genes. This normalization is applied by using Formula 1, where *c *is the count data, *m *is the mean of the sum of the housekeeping genes across all samples, and *s *is the sum of the housekeeping genes for a given sample.

*Option 2*: Normalize to the entire miRNA sample. The normalization is employed by using Formula 1, where *c *is the count data, *m *is the mean of the sums of the counts for each sample, and *s *is the sum of all of the counts for each sample.

*Option 3*: Normalize to the highest miRNA in an assayed sample. Unlike Option 2, which uses all of the genes, this option normalizes the data to only the highest miRNA counts (default top 75) for a given sample (default sample 1) for the normalization factor.

### Differential expression

After all of the relevant correction and normalization steps have been applied, statistical tests are applied to determine differentially expressed genes. If the data is assumed to be distributed normally, a t-test (for two conditions) or one-way ANOVA (for three or more conditions) can be performed on the sample content-normalized data to determine statistical significance. If a negative binomial distribution is assumed, DESeq (for two conditions, using the DESeq R library) or a one-way ANODEV (for three or more conditions, using the DESeq R library) can be used on the DESeq normalized to determine statistical significance. When estimating the dispersion factors in DESeq used for normalization, different parameters are implemented. If only single replicates are used for each condition, the blind method is used to estimate dispersions with a fit-only sharing mode. If any condition has only two biological replicates, the pooled method is used with a fit-only sharing mode. If neither of the previous two conditions is met, a pooled method with a maximum sharing mode is used to estimate the dispersions.

### Heatmap generation and downloadable output

**Figure 1:**
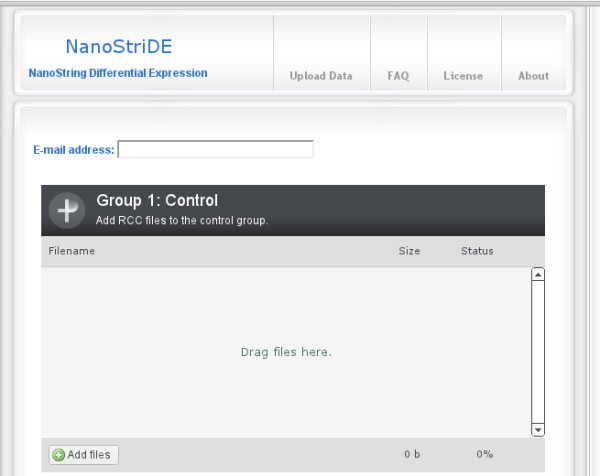
**Screenshot of NanoStriDE Web Interface**. This screenshot of the web interface of NanoStriDE shows the interface used to quickly select and upload multiple NanoString raw data files to the server for processing.

**Figure 2:**
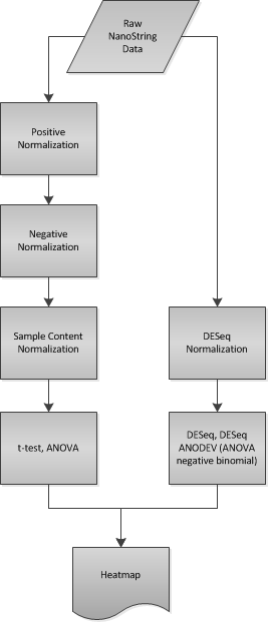
**NanoStriDE Workflow**. The workflow of normalization steps and heatmap generation is depicted. Correction and normalization steps process the raw data first after which a user selected statistical test is used to determine significant genes to include in the heatmap generation. Not shown in this diagram is that every process depicted between the raw data to the heatmap generation has the intermediate data recorded and returned to the user in the downloadable output.

Only a subset of genes whose change in expression level are statistically significant are selected for visualization in a heatmap. A user-defined p-value threshold applied to the results from differential expression analysis removes any genes with p-values at or above the cutoff value. This cutoff is applied to either the p-value or to an adjusted p-value as specified by the user. The Bonferroni, Holm, Hochberg, Hommel, Benjamini & Hochberg, and Benjamini & Yekutieli p-value corrections are available as valid adjusted p-value options. A mean cutoff is applied using the subset from the p-value cutoff, removing any genes with a final normalized count mean at or below the cutoff value. If there are two or more remaining genes, a heatmap representative of the differential expression analysis is generated on a log scale across the rows. Refer to Figure 1 for a view of the interface used to upload multiple raw data files and Figure 2 for an overview of the normalization, differential expression analysis, and heatmap generation steps.

## Results

NanoStriDE is configured to be easily accessible to biologists who employ nCounter to perform sophisticated experiments, so that they may complete their analyses without specialized training in biostatistics. A user's guide outlining how to use the website as well as details on all options and warnings is available on the NanoStriDE website. The analyzed data from a completed job can be downloaded in a number of stages and formats from NanoStriDE. Corrected raw, normalized data, results of differential expression analysis for all probes, user settings, and customized readme file are available for download in text format.

## Conclusions

The NanoStriDE web application was developed to assist biologists with performing and interpreting differential expression analaysis from NanoString nCounter system data. The high resolution heatmaps produced by NanoStriDE are suitable for use as figures in publications. If the user wishes to independently perform differential analysis on the data, the normalized data is additionally provided in the output of the completed job. NanoStriDE was designed for ease of use allowing biologists with limited computational experience to perform sophisticated differential expression analysis.

## Availability and requirements

Project Name: NanoStriDE

Project Homepage: http://nanostride.soe.ucsc.edu

Operating System: Platform independent

Programming Language: Perl, PHP, R

Other Requirements: Modern HTML 5 compliant web browser (e.g. IE 9, Firefox 8, Chrome 15, Opera 11)

License: NanoStriDE is freely available under the GPL v2 license and can be found on the NanoStriDE web page on the license page.

## Authors' contributions

CDB designed and implemented the web interface and the corresponding backend scripts required to run the pipeline. HJK and MG participated in its design and helped to draft the manuscript. NP conceived of the study, participated in its coordination and helped to draft the manuscript. All authors read and approved the final manuscript.
